# Common pitfalls in renal mass evaluation: a practical guide

**DOI:** 10.1590/0100-3984.2018.0007

**Published:** 2019

**Authors:** Layra Ribeiro de Sousa Leão, Thais Caldara Mussi, Fernando Ide Yamauchi, Ronaldo Hueb Baroni

**Affiliations:** 1 Department of Radiology and Diagnostic Imaging, Hospital Israelita Albert Einstein, São Paulo, SP, Brazil.

**Keywords:** Kidney neoplasms/diagnostic imaging, Magnetic resonance imaging, Ultrasonography, Computed tomography, Neoplasias renais/diagnóstico por imagem, Ressonância magnética, Ultrassonografia, Tomografia computadorizada

## Abstract

More than half of patients over 50 years of age have had at least one focal renal lesion detected as an incidental finding during an ultrasound, computed tomography, or magnetic resonance imaging examination. Although the majority of such lesions can be easily detected and correctly characterized, misdiagnoses may occur and are often related to methodological limitations, inappropriate imaging protocols, or misinterpretation. This pictorial essay provides recommendations on how to recognize benign and malignant renal processes that can be potentially missed or mischaracterized in imaging studies.

## Abstract (pt)

Mais da metade dos pacientes com mais de 50 anos de idade pode ter pelo menos uma lesão renal focal detectada de modo incidental em estudos de imagem, como ultrassonografia, tomografia computadorizada ou ressonância magnética. Embora a maioria dessas lesões possa ser facilmente caracterizada, erros de diagnóstico podem ocorrer e geralmente estão relacionados a limitações de métodos, protocolos de imagem inadequados e interpretação incorreta. Este ensaio iconográfico aborda recomendações sobre como reconhecer e interpretar lesões focais renais em estudos de imagem.

## INTRODUCTION

Detection of incidental renal masses has grown exponentially due to widespread use of ultrasound, computed tomography (CT), and magnetic resonance imaging (MRI) for a variety of indications^(^^1^^)^. Despite several technical improvements, there are pitfalls in the detection and characterization of such masses because of inherent methodological limitations or the use of inappropriate protocols^(^^2^^)^. Misinterpretation is another source of pitfalls, and recognizing potentially confounding situations allows radiologists to avoid misdiagnosis in the evaluation of renal masses.

**Figure 1:**
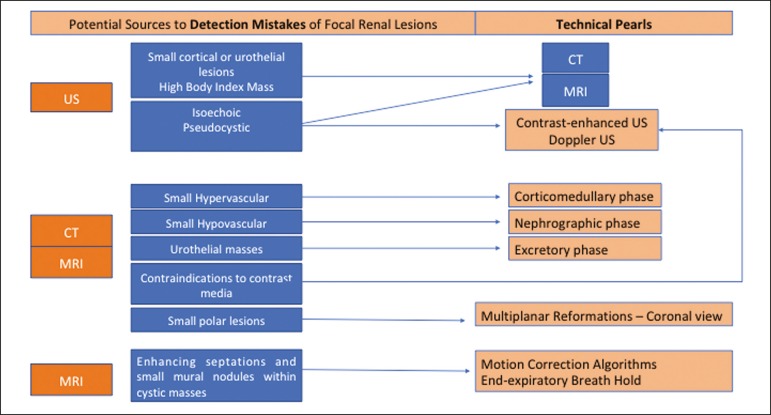
Proposed algorithm to avoid errors in detection in the evaluation of renal masses.

**Figure 2:**
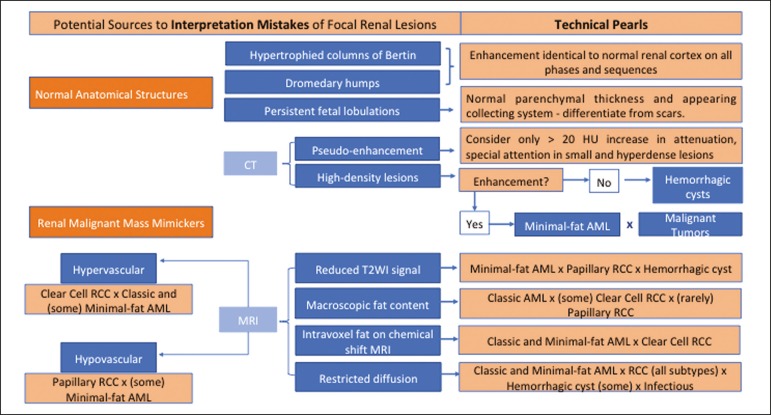
Proposed algorithm to avoid errors in interpretation in the evaluation of renal masses.

This pictorial essay illustrates several cases of misdiagnosis or near-missed diagnosis of renal lesions on ultrasound, CT, and MRI, obtained for review from our database. Cases were divided into two categories-errors in detection and errors in interpretation-and we propose two algorithms to avoid those pitfalls (Figures 1 and 2, respectively).

## ERRORS IN DETECTION

Ultrasound, CT, and MRI are the most common modalities for the detection and characterization of renal lesions. The use of inappropriate protocols can lead to pitfalls, as can suboptimal image quality^(^^2^^)^.

### Ultrasound

**Figure 3:**
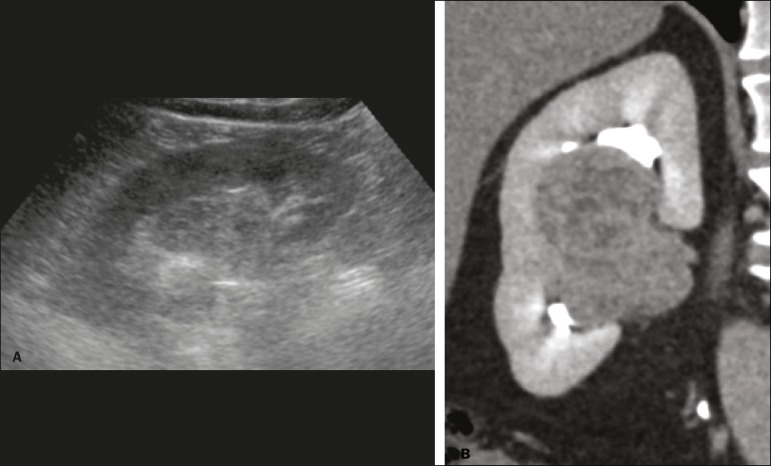
Renal masses missed on initial ultrasound and detected on subsequent CT. A: Right renal tumor missed on initial ultrasound and mistaken for the renal sinus. B: CT clearly shows a heterogeneous mass projecting into the renal sinus. The final diagnosis was clear cell renal cell carcinoma.

Ultrasound is usually the primary imaging modality for the detection of renal masses and is useful in determining whether or not the lesion is cystic in nature. In some cases, ultrasound can also demonstrate the internal characteristics of complex cystic masses, such as septa, calcifications, and mural nodules. When the mass presents those characteristics, CT or MRI should be performed for a complete evaluation. The advantages of ultrasound, in comparison with cross-sectional imaging methods, include its wide availability and lower cost, as well as the fact that it does not require the use of ionizing radiation or nephrotoxic intravenous contrast agents^(^^2^^,^^3^^)^. However, ultrasound is operator-dependent and the detection of renal lesions may be impaired in patients with a high body mass index, interposition of bowel gas, and small or isoechoic tumors (Figure 3).

**Figure 4:**
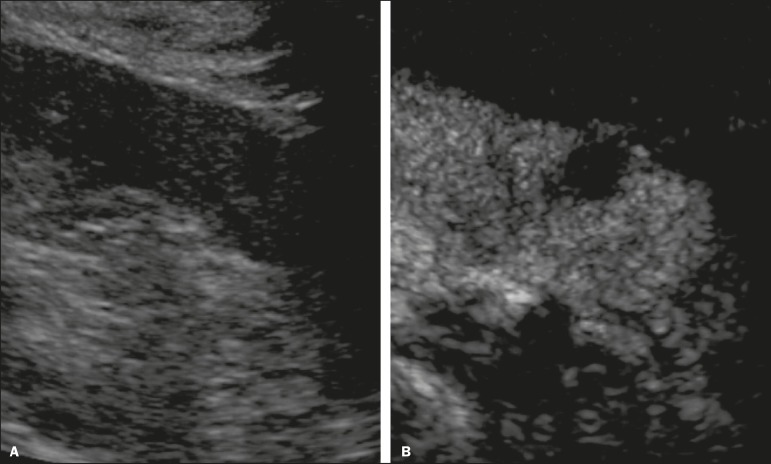
Contrast-enhanced ultrasound images. A: An isoechoic mass in the periphery of the left kidney was initially missed. B: Contrast-enhanced ultrasound showing a small hypovascular mass in the posterior aspect of the left kidney. Histopathology confirmed a papillary renal cell carcinoma.

The most important step to avoid errors in detection is to ensure visualization of the entire parenchyma, perinephric space, and renal sinus. Color Doppler imaging may help identify an area of abnormal parenchymal vascularization or the displacement of normal renal vessels by a renal mass. The use of contrast-enhanced ultrasound in the evaluation of focal renal lesions is promising, enabling characterization of enhancement in different structures, such as vessels, septations, mural nodules, and hypovascular lesions (Figure 4). Because ultrasound contrast agents are not nephrotoxic or hepatotoxic, they may be an alternative for patients in whom the use of CT and MR contrast media is contraindicated.

### CT

CT has higher sensitivity for the detection of renal lesions than does conventional ultrasound. The diagnostic accuracy of CT is reported to be as high as 95%, and CT is the most widely used imaging method. Current imaging techniques allow rapid acquisition of thin-slice images during a short breath hold, minimizing artifacts due to motion or misregistration. Various morphological features of renal masses can be evaluated by CT, including their internal content (calcifications, fat, areas of necrosis, septations, mural nodules, and the cystic component) and enhancement. 

**Figure 5:**
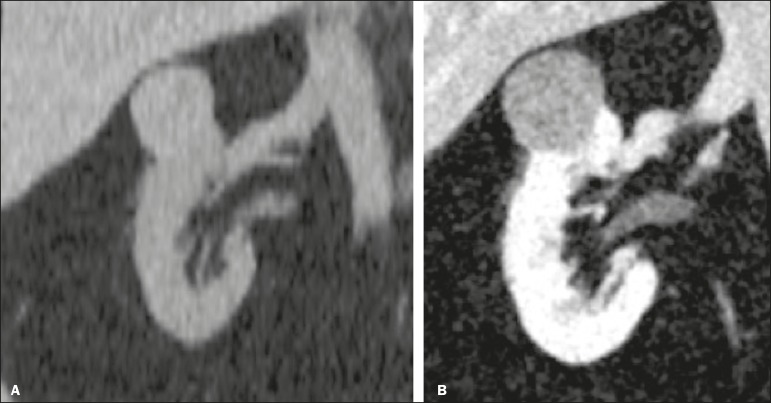
The importance of an appropriate protocol and the use of intravenous contrast. A: Unenhanced CT showing a hyperattenuating renal nodule that could be either a hemorrhagic cyst or a solid mass. B: After intravenous contrast administration, there was discrete but measurable enhancement (37-62 HU), confirming the diagnosis of a solid renal mass.

An appropriate CT protocol includes an unenhanced phase followed by corticomedullary, nephrographic, and excretory phases (Table 1). Unenhanced images allow detection of calcifications, hemorrhagic material, and fat component, as well as serving as a baseline for quantification of the subsequent enhancement. In the corticomedullary phase, images are acquired at 40-60 s after contrast administration and provide the maximum differentiation between the cortex and medulla^(^^2^^)^, facilitating the detection of small hypervascular renal masses as well as allowing the assessment of tumor vascularity, renal artery segmentation, and potential anatomical variations, the knowledge of which is useful for surgical planning. In the nephrographic phase, images are acquired at 80-100 s after contrast administration. Although the cortex and medulla exhibit similar enhancement in this phase, it is easier to detect small renal masses, especially hypovascular ones (Figure 5). In the excretory phase, images are acquired at 5-7 min after contrast administration and helps characterize the relationship between the mass and renal collecting system, as well as facilitate the differentiation between parenchymal masses and urothelial masses.

**Table 1:** Multidetector CT parameters for the four acquisition phases.

Parameter	Phase			
	Unenhanced	Corticomedullary	Nephrographic	Excretory
Scan delay	Not applicable	Fixed, 40-60 s after contrast	Fixed, 80-100 s after contrast	Fixed, 5-7 min after contrast
		injection	injection	injection
Detector configuration	0.5 mm × 80 mm	0.5 mm × 80 mm	0.5 mm × 80 mm	0.5 mm × 80 mm
Reconstruction slice thickness	1 mm	2 mm	2 mm	3 mm

An appropriate CT protocol for the detection of renal lesions should focus on optimizing not only multi-phase image acquisition but also narrow collimation, reduced pitch, and thin overlapping reconstructions. In addition, multiplanar reconstructions should be routinely performed, given that minor contour deformation and polar lesions can be difficult to identify on axial images^(^^4^^)^.

### MRI

Compared with CT, MRI has better contrast resolution and does not expose patients to ionizing radiation^(^^5^^)^. When the CT findings are inconclusive, the patient can be better evaluated by MRI, as can pediatric patients, pregnant patients, and patients for whom the use of contrast media in contraindicated^(^^6^^,^^7^^)^.

At our institution, the MRI protocol for renal masses includes the following (Table 2): T2-weighted imaging (T2WI); chemical shift imaging, involving in-phase and out-of-phase T1-weighted imaging (T1WI); diffusion-weighted imaging (DWI); coronal fat-suppressed T1WI with gradient-recalled echo sequences performed before and after administration of intravenous gadolinium contrast; and post-processed images with digital subtraction. Multiplanar imaging is also recommended, because small renal nodules may be better depicted in a particular imaging plane depending on the orientation of the kidney.

**Table 2:** Multiparametric MRI protocol.

					Slice			Reconstruction
				FOV	thickness		TR/TE	slice thickness
Sequence	Imaging plane	Physiology	Volumetry	(cm)	(mm)	Matrix	(ms)	(mm)
AX T2-WI fat-suppressed	Axial	Respiratory-triggered	2D	34	6	-	79/283-14,000	1
DWI (b value = 0/50 s/mm^2^)	Axial	Respiratory-triggered	2D	34	6	224 × 256	1.0/13,332	1
DWI (b value = 400/800 s/mm^2^)	Axial	Respiratory-triggered	2D	34	6	224 × 256	15,485	1
Single-shot fast spin-echo T2WI	Coronal	Breath-hold	2D	38	5	256 × 224	120/minimum	1
T2WI	Axial	Breath-hold	2D	34	5	256 × 192	160/4100	1
Chemical shift (in-phase + out-of-phase) T1WI	Axial	Breath-hold	2D	34	03/08/19	256 × 192	Minimum- 4.2/6.2	1
Chemical shift (in-phase + out-of-phase) T1WI	Coronal	Breath-hold	2D	30	5	256 × 160	2.2; 4.5/230	1
Unenhanced T1WI fat-suppressed GRE	Axial + coronal	Breath-hold	3D	38	03/08/19	256 × 224	1.4/3.3	-
Post-gadolinium dynamic fat-suppressed GRE	Coronal + axial	Breath-hold	3D	38	3.8/3.4	256 × 224	1.9/4.4	-
	(delayed)				(delayed)			

Subtraction imaging is considered a problem-solving tool when evaluating subtle enhancement on MRI, particularly when a lesion has hemorrhagic or proteinaceous contents that generate high signal intensity on pre-contrast T1WI^(^^8^^)^. Densely calcified and heterogeneous hemorrhagic masses are better evaluated on MRI because the pseudo-enhancement that occurs on CT, due to the beam hardening effect, does not occur on MRI.

There are several image acquisition-related pitfalls that can result in errors in detection, especially in less experienced readers. Respiratory motion artifacts can make it difficult to detect enhancing septations and small mural nodules within cystic masses. In addition, variations in breath-hold sequences may cause misregistration artifacts on post-processing with digital subtraction. In such situations, user-defined regions of interest (ROIs) may help characterize enhancement based on the relative increase in signal intensity between pre- and post-contrast images (more than 15-20%), assuming that the same acquisition parameters are used^(^^9^^)^. Motion artifacts can be minimized by employing motion correction algorithms and an end-expiratory breath hold^(^^2^^,^^5^^)^.

## ERRORS IN INTERPRETATION

**Figure 6:**
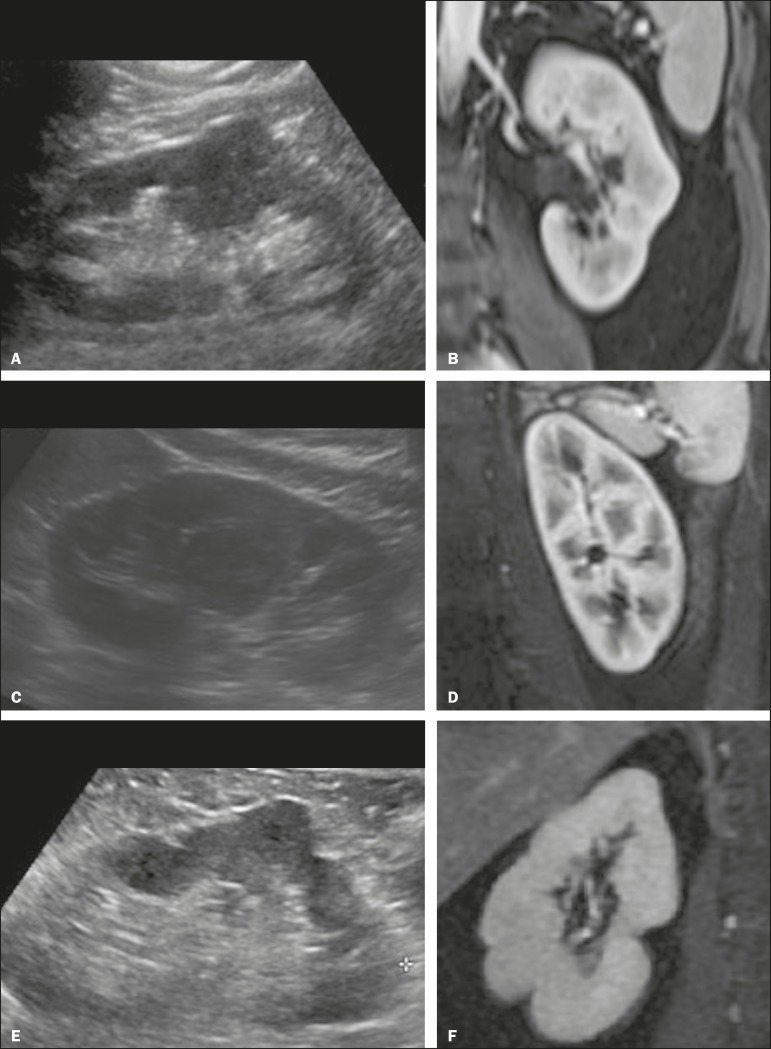
Dromedary hump, hypertrophied column of Bertin, and persistent fetal lobulation. A: Ultrasound showing an external bulge on the lateral border of the left kidney, reported as a possible nodule. B: CT confirmed a renal lobulation (dromedary hump). C: Sagittal ultrasound showing a mass-like area in the left kidney. D: Coronal contrast-enhanced T1-weighted MRI showing enhancement similar to that of the adjacent normal renal cortex, an aspect consistent with hypertrophied column of Bertin. E: Ultrasound showing an external bulge on the right kidney, misinterpreted as a renal nodule. F: Contrast-enhanced coronal T1-weighted MRI showing lobulated contours, consistent with persistent fetal lobulation. Note the normal parenchymal thickness and normal appearance of the renal collecting system, features that are helpful in differentiation this from scarring.

Normal renal structures may mimic cystic or solid neoplasm on imaging studies. On ultrasound, extensions of renal cortical tissue between the renal pyramids (hypertrophied column of Bertin) may appear as a solid mass projecting into the renal sinus^(^^10^^)^. Similarly, normal renal parenchyma adjacent to scarring can be misinterpreted as a solid mass. These pitfalls are easily avoided on CT and MRI, because the attenuation, signal intensity, and enhancement are identical to those of normal renal cortical tissue in all phases and sequences^(^^11^^)^, as shown in Figures 6A and 6B.

A dromedary hump, albeit a normal variant of the renal contour, can mimic a renal mass on ultrasound. It is defined as a focal bulge on the lateral border of the left kidney and is caused by the splenic impression onto the superolateral left kidney. It is easily recognized on CT and MRI because it exhibits the same attenuation, signal intensity, and enhancement as the surrounding normal renal parenchyma (Figures 6C and 6D).

Persistent fetal lobulation of the kidney may be mistaken for scarring. Small indentations of the renal cortex without cortical thinning, abnormal enhancement, and retracted underlying collecting system are clues to a diagnosis of persistent fetal lobulation (Figures 6E and 6F).

**Figure 7:**
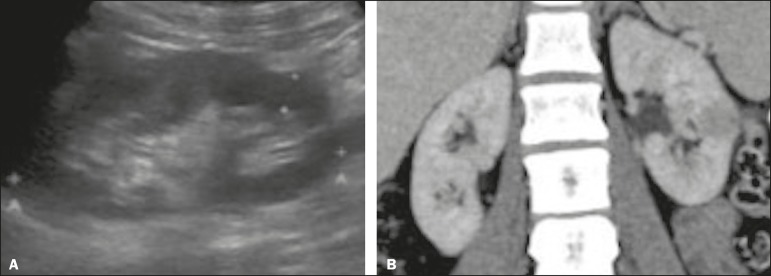
Focal pyelonephritis. Ultrasound (A) and CT (B) of the left kidney showing a well-defined, focal, hypoechoic mass-like lesion. Given that the patient had a history of urinary tract infection, this finding is most consistent with focal pyelonephritis. Ultrasound findings were normal after 4 weeks of antibiotic treatment.

Inflammatory masses and vascular structures can have an appearance similar to that of a neoplasm on imaging exams, and the clinical context is helpful to distinguish these entities. When clinical findings of infection are not present, focal pyelonephritis can mimic solid neoplasm or, in the presence of an abscess, a complex cystic neoplasm (Figure 7). In such cases, a poorly defined interface between the infection and the renal parenchyma can be diagnostic, as can edema or asymmetric perinephric stranding.

**Figure 8:**
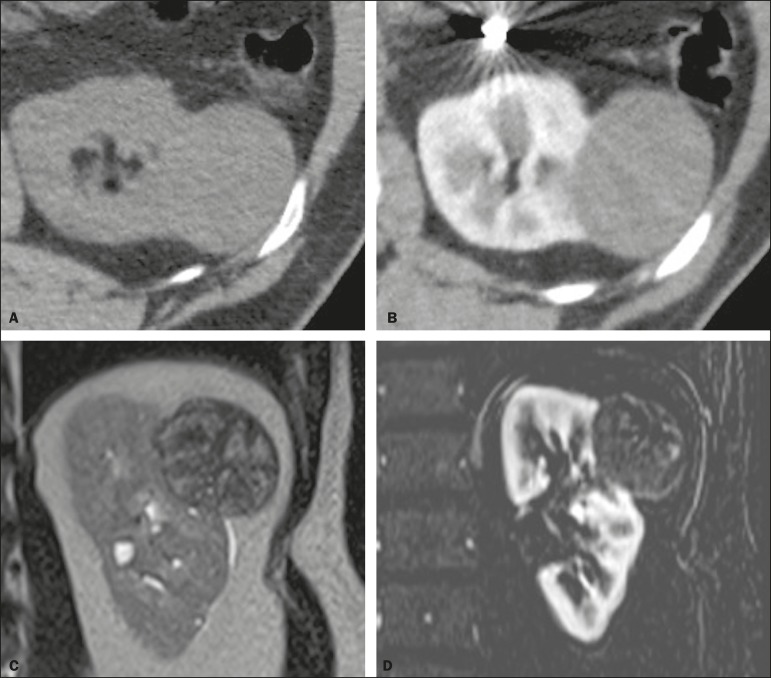
Cystic versus hypovascular mass. A,B: CT showing a hypoattenuating lesion, initially mistaken for a simple cyst, in the left kidney. C,D: MRI showing an exophytic heterogeneous mass with predominantly low signal intensity on T2WI (C). Images with digital subtraction (D) may help confirm the presence of solid enhancing components. Histopathology confirmed a papillary renal cell carcinoma.

Most malignant renal neoplasms are clear cell renal cell carcinomas and demonstrate avid enhancement after intravenous administration of contrast media, making it easy to identify them on CT. However, the papillary variant of renal cell carcinoma usually presents as hypovascular lesions that can be misdiagnosed as renal cysts on CT (Figure 8). The visual analysis is challenging and ROIs should always be placed over the lesions to confirm enhancement (> 20 HU). When the CT findings are inconclusive, MRI should be performed, given its higher contrast resolution.

## CONCLUSION

There are numerous pitfalls in the evaluation of renal lesions, including errors in detection and interpretation. Although ultrasound is usually the first-line imaging modality, it has lower sensitivity for the detection of renal lesions than do CT and MRI. Small lesions and hemorrhagic lesions may be difficult to evaluate on CT, and an ROI should be placed over every renal mass evaluated. All indeterminate renal lesions should be further evaluated with MRI, and the images should undergo post-processing with digital subtraction.

## References

[1] Chow WH, Devesa SS, Warren JL (1999). Rising incidence of renal cell cancer in the United
States. JAMA.

[2] Katabathina VS, Shiao J, Flaherty E (2016). Cross-sectional imaging of renal masses: image
interpretation-related potential pitfalls and possible
solutions. Semin Roentgenol.

[3] Stakhovskyi O, Yap SA, Leveridge M (2011). Small renal mass: what the urologist needs to know for treatment
planning and assessment of treatment results. AJR Am J Roentgenol.

[4] Johnson PT, Horton KM, Fishman EK (2010). Optimizing detectability of renal pathology with MDCT: protocols,
pearls, and pitfalls. AJR Am J Roentgenol.

[5] Ramamurthy NK, Moosavi B, McInnes MD (2015). Multiparametric MRI of solid renal masses: pearls and
pitfalls. Clin Radiol.

[6] Heilbrun ME, Remer EM, Casalino DD (2015). ACR appropriateness criteria indeterminate renal
mass. J Am Coll Radiol.

[7] Miranda CLVM, Sousa CSM, Bastos BB (2018). Giant renal angiomyolipomas in a patient with tuberous
sclerosis. Radiol Bras.

[8] Pedrosa I, Sun MR, Spencer M (2008). MR imaging of renal masses: correlation with findings at surgery
and pathologic analysis. Radiographics.

[9] Hecht EM, Israel GM, Krinsky GA (2004). Renal masses: quantitative analysis of enhancement with signal
intensity measurements versus qualitative analysis of enhancement with image
subtraction for diagnosing malignancy at MR imaging. Radiology.

[10] Son J, Lee EY, Restrepo R (2012). Focal renal lesions in pediatric patients. AJR Am J Roentgenol.

[11] Bradley AJ, Lim YY, Singh FM (2011). Imaging features, follow-up, and management of incidentally
detected renal lesions. Clin Radiol.

